# Insecticide resistance in *Culex quinquefasciatus* Say, 1823 in Brazil: a review

**DOI:** 10.1186/s13071-019-3850-8

**Published:** 2019-12-18

**Authors:** Ramon Pereira Lopes, José Bento Pereira Lima, Ademir Jesus Martins

**Affiliations:** 1 Laboratório de Fisiologia e Controle de Artrópodes Vetores, Instituto Oswaldo Cruz, Fiocruz, Rio de Janeiro, Brazil; 2 Instituto Nacional de Ciência e Tecnologia em Entomologia Molecular, Universidade Federal do Rio de Janeiro, Rio de Janeiro, Brazil

**Keywords:** Vector control, Southern house mosquito, Insecticide resistance monitoring, Urban vector, Filarial vector

## Abstract

*Culex quinquefasciatus* is a successful invasive species broadly distributed in subtropical regions, including Brazil. It is an extremely annoying mosquito due to its nocturnal biting behavior, in high-density populations and it is a potential bridge between sylvatic arbovirus from birds to man in urban territories. Herein, we present a review concerning the methods of chemical control employed against *Cx. quinquefasciatus* in Brazil since the 1950’s and insecticide resistance data registered in the literature. As there is no specific national programme for *Cx. quinquefasciatus* control in Brazil, the selection of insecticide resistance is likely due in part to the well-designed chemical campaigns against *Aedes aegypti* and the elevated employment of insecticides by households and private companies. There are very few publications about insecticide resistance in *Cx. quinquefasciatus* from Brazil when compared to *Ae. aegypti*. Nevertheless, resistance to organophosphates, carbamate, DDT, pyrethroids and biolarvicides has been registered in *Cx. quinquefasciatus* populations from distinct localities of the country. Concerning physiological mechanisms selected for resistance, distinct patterns of esterases, as well as mutations in the acetylcholinesterase (*ace-1*) and voltage-gated sodium channel (*Na*_*V*_) genes, have been identified in natural populations. Given environmental changes and socioeconomical issues in the cities, in recent years we have been experiencing an increase in the number of disease cases caused by arboviruses, which may involve *Cx. quinquefasciatus* participation as a key vector. It is urgent to better understand the efficiency and susceptibility status to insecticides, as well as the genetic background of known resistant mechanisms already present in *Cx. quinquefasciatus* populations for an effective and rapid chemical control when eventually required.

## Background

*Culex quinquefasciatus* Say, 1823 (Diptera: Culicidae) known as the southern house mosquito, is a subtropical mosquito belonging to the complex *Culex pipiens*, present in the Americas, Australia, Asia, Africa, Middle East and New Zealand, as well as being broadly distributed in Brazil [1, 2]. Amongst the several species in genus *Culex* registered in Brazil [3], *Cx. quinquefasciatus* stands out as the most abundant and anthropophilic species [4]. In addition to the considerable discomfort caused by the nocturnal biting behavior, *Cx. quinquefasciatus* is the main vector of several pathogens, especially including the nematode *Wulchereria bancrofti* (agent of bancroftian filariasis) and the West Nile virus [5, 6]. As mosquitoes from the complex *Cx. pipiens* feed both on human and bird blood, they may potentially transport sylvatic arboviruses from migratory birds to man in urban territories [7]. This mosquito is also a potential vector of the arboviruses responsible for the Rift Valley fever [8] and Saint Louis encephalitis [9]. In the recent Brazilian Zika outbreak, samples of *Cx. quinquefasciatus* from urban environments were detected to be infected with ZIKV, suggesting participation in a new cycle of this emergent arbovirus in some regions [10, 11]. Similarly, its role has also been implied in the transmission of the emergent Mayaro virus in urban centers [12]. Other studies considering systems of infection in the laboratory demonstrated that *Cx. quinquefasciatus* would also be competent to transmit the protozoan *Plasmodium relictum* (agent of bird malaria) [13] and *Hepatozoon breinli*, an intracellular parasite infecting birds, reptiles, amphibians and rodents [14]. An extensive list of viruses, protozoans and nematodes isolated from *Cx. quinquefasciatus* under natural and laboratory conditions can be found elsewhere [15].

In the absence of effective vaccines available against most of the *Culex-*transmitted pathogens, the best strategy to avoid transmission relies on the chemical control of the mosquito [16]. At the end of the last century, the World Health Organization (WHO) launched a manual focused on mosquito vector control, including *Culex* spp., highlighting the necessity of measures to prevent their reproduction and dispersion [17]. Although *Culex* spp. females preferentially lay their eggs in collections of water, either stagnant or gentle flow, rich in organic matter, *Cx. quinquefasciatus* is very opportunistic so that any permanent or temporary collection of water may serve as a potential breading site for their larvae [1, 18]. Therefore, vector control planning has to focus on breeding-site elimination or treatment by improving the basic sanitary infrastructure of water supply and waste destination, as well as activities to promote community engagement within an environmental agenda. However, given the accelerated and disorganized process of urbanization in the last decades, especially in the tropical, low-income countries, these tasks are too complex to be fully achieved. Additionally, even in well-developed regions, the density of these mosquitoes may be positively correlated with seasonal high temperatures [19, 20]. In this scenario, chemical larvicides or polystyrene granules can be applied to water collections. Insecticide residual spraying (IRS) in the interior of the houses is generally not effective against *Cx. quinquefasciatus* given its habit of posing on substrates generally not treated with insecticide, such as cloth, curtains and other suspended fabrics, instead of resting on the walls and ceiling [17].

The first official actions in Brazil specifically targeting *Cx. quinquefasciatus* based on chemical control were during the years 1951–1955, as a first phase of a governmental campaign to control Bancroftian filariasis [21]. This campaign was coordinated by the National Service of Malaria, opting the use of hexachlorobenzene (BHC), dichlorodiphenyltrichloroethane (DDT) and dieldrin as residual action insecticides [22]. The second phase of this campaign was initiated in 1956, with the creation of the National Department of Rural Endemics (DNERu). By 1960, 120,339 midguts were dissected from female mosquitoes caught in filariasis endemic areas, which recognized *Cx. quinquefasciatus* (at that time named *Cx. pipiens fatigans*) as the main vector in the country [21]. In the following decade, Brazilian national campaigns against filariasis were coordinated by the Superintendence of Public Health Campaigns (SUCAM). These campaigns aimed at eradicating or controlling the filariasis transmission in endemic areas by treating committed persons with the chemotherapy diethylcarbamazine, as well as decreasing the density of the mosquito by improving the sanitary infrastructure and applying residual insecticides (BHC and dieldrin) against both larvae and adult stages of *Cx. quinquefasciatus* [22].

The employment of residual insecticides (BHC, DDT and dieldrin) for controlling adult mosquitoes was incipiently effective. Nevertheless, they became ineffective, their use being suspended [22]. Given the lack of an efficient adulticide together with the high cost of larvicide applications in the breading sites, the chemical treatment was discontinued and the national programmes centralized their actions on the treatment of human cases and health educational programmes [22]. Currently, the Brazilian Ministry of Health (MoH) acquires the insecticides and provides them to the states, which supply the municipalities. In turn, the municipalities have autonomy to complement alternative compounds in their territories, as long as they are approved by the WHO and the Brazilian National Agency of Sanitary Surveillance (ANVISA) [23]. There is no specific national programme for combating *Culex*, as most of the governmental actions against this mosquito are a side-effect of the well-structured programme for *Aedes aegypti* control. In this sense, most of the insecticide selection pressure geared toward *Cx. quinquefasciatus* populations in Brazil is substantially derived from that targeting *Ae. aegypti* [24].

In the last three decades the main larvicides utilized in Brazil under national scale against *Ae. aegypti* have been the organophosphate temephos, followed by the IGRs (insect growth regulators class) diflubenzuron, novaluron and more recently, pyriproxyfen. Pyrethroids were adopted as adulticides from 2000 until 2013 when the organophosphate malathion began to be implemented, as the only permissible alternative after reports that pyrethroid resistance in *Ae. aegypti* was apparent all over the country [23, 25]. Nevertheless, commercial pyrethroids have intensively been sprayed inside the dwellings as well as under thermo-fogging or ultra-low volume in the peri-domicile by private companies. In addition to neurotoxic insecticides, as recommended by the WHO [26], the bacterium *Lysinibacillus sphaericus* (*Lbs*), previously known as *Bacillus sphaericus* (*Bs*) [27], is also indicated and largely enlisted as a biolarvicide for *Culex* control [28]. *Lysinibacillus sphaericus* began to be exploited on a large scale for *Culex* control in Brazil since 1989, ever since used as a larvicide by the Filariasis Elimination Programme in Recife/PE and on the border of the Pinheiro River in São Paulo [29, 30].

Likewise, as with *Aedes* and *Anopheles* mosquitoes, the exacerbated use of insecticides has been selecting resistant *Cx. quinquefasciatus* populations around the world [31–34]. Resistance to insecticides is a multi-factorial genetic trait, preceding insecticide exposure. Normally, the frequency of resistant insects in natural populations is very low in an environment free of insecticides, i.e. without a selection pressure. Hence, the continuous application of insecticides favorably selects the resistant individuals, while those susceptible are progressively eliminated, reducing the genetic variability of the target population [35]. Depending on the intensity of the selection pressure over genetically well-structured and isolate populations, resistance may become irreversible due to the lack of susceptible mosquitoes to contribute their genes to the next generations, where migration among other populations is absent or very low [36]. In Brazil, in addition to the chemicals employed in governmental campaigns, the uncontrolled application by households increases during arbovirus outbreaks and also when targeting *Culex* itself due to its usual high densities and annoying nocturnal biting behavior [37, 38]. As the odor of pyrethroids is less noxious to the people, this class of insecticide is largely preferred [39, 40]. There is evidence that this excessive household use of chemicals is the main factor contributing to pyrethroid resistance selection in *Ae. aegypti* [41, 42], *Cx. quinquefasciatus* populations as such being likely to experience a similar phenomenon.

There are four main classes of mechanisms attributed to resistance in a mosquito population: behavioral changes, decrease of cuticular penetration, increase in the metabolic detoxification and alteration in the insecticide target-molecule, these two latter mechanisms being the mostly molecularly elucidated [43–45]. An increase in the metabolic detoxification may occur due to an increase in the detoxification power, generally related to the classes of enzymes esterases, glutathione S-transferases (GSTs) and multi-function oxidases P450s, which are able to modify or break up the insecticide molecules before they reach their target. In turn, target-site alterations inhibit the interaction between the insecticide and its action target molecules, rendering the insecticide less effective or even ineffective [46].

**Fig. 1:**
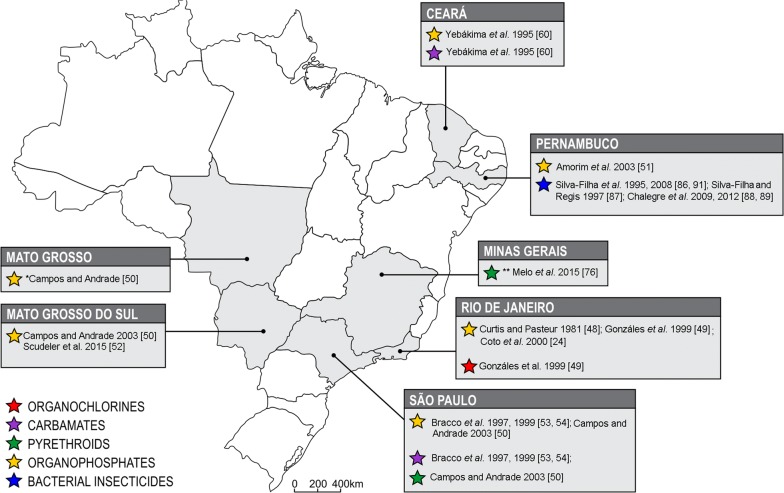
Representation of insecticide resistance records in *Culex quinquefasciatus* from Brazil *Tolerance detection to organophosphates. **Laboratory strain

In 2011, the Brazilian MoH launched a surveillance and control methods guide against *Cx. quinquefasciatus*, recommending the use of neurotoxic (pyrethroids, carbamates or organophosphates) and IGR compounds (juvenile hormones analogues and benzophenil ureas, as chitin synthesis inhibitors) in conjunction with the biolarvicide *Lbs* [5], very similarly as indicated against *Ae. aegypti*. The compounds currently indicated by the MoH are: organophosphates and pyrethroids for adult control and spinosyns; bacterium biolarvicides; benzophenilureas; juvenile hormone analogues; and organophosphates against larvae [47]. Nevertheless, there are some reports of *Cx. quinquefasciatus* populations resistant to some of these compounds in the country (see Fig. 1). A list with insecticide resistance data available in the literature, including susceptibility tests, biochemical and molecular assays, distributed per region and year are provided in Table 1.

**Table 1:** List of insecticide resistance data on *Culex quinquefasciatus* field-collected in Brazil

Macro Region	State	Region	Year	Insecticide	Bioassay	Diagnostic-dose		Biochemical and molecular assays		
					Dose–response					
					RR_50_ (LC_50_ or LT_50_) or RR_95_ (LC_95_)	Dose (%mortality)	Status^d^	Method	Detected mechanism	Reference
North	Amazonas	Manaus	1978	Chlorpyriphos	1.3 (0.0008)	–	S	–	–	Curtis & Pasteur [48]
North-East	Pernambuco	Recife	1995	–	–	–	–	Sequencing	G119S ace-1^R^	Weill et al. [64]
		Coque^a^	1991–1993	*Lysinibacillus sphaericus*	9.7 (0.0367)	–	Low resistance	Binding experiments	Slight decrease in the receptor concentration	Silva-Filha et al. [86]
			1991–1993	*Bacillus thuringiensis* svar*. israelensis*	2.0 (0.01)	–	S	–	–	Silva-Filha et al. [86]
			1996	*Lysinibacillus sphaericus*	1.3–7.3 (0.0013–0.0313)	–	Low resistance	–	–	Silva-Filha & Regis [87]
			1996	*Bacillus thuringiensis* svar*. israelensis*	1.0 (0.009)	–	S	–	–	Silva-Filha & Regis [87]
		Água Fria^a^	2005–2010	*Lysinibacillus sphaericus*	2.7–8.6 (0.008–0.024)	–	Low resistance	AS-PCR	cqm1_REC_ (0.033–0.055)^e^; cqm1_REC-D25_ (0.002)^e^	Silva-Filha et al.; Chalegre et al. [88, 89, 91]
			2009–2010	Temephos	1 (0.006)	–	S	–	–	Amorim et al. [51]
		Azeitona^a^	1999	*Lysinibacillus sphaericus*	–	–	–	AS-PCR	cqm1_REC_ (0.002)^e^; cqm1_REC-D16_ (0.006)^e^	Chalegre et al. [89]
		Roda de Fogo^a^	1999	*Lysinibacillus sphaericus*	–	–	–	AS-PCR	cqm1_REC_ (0.017)^e^	Chalegre et al. [89]
		Fazenda Nova	2007	*Lysinibacillus sphaericus*	3.7 (0.011)	–	S	AS-PCR	cqm1_REC_ (0.0029)^e^	Chalegre et al. [88]
		Peixinhos^a^	2007	*Lysinibacillus sphaericus*	4 (0.012)	–	S	AS-PCR	cqm1_REC_ (0.0061)^e^	Chalegre et al. [88]
			2009–2010	Temephos	1 (0.006)	–	S	–	–	Amorim et al. [51]
		Jaboatão dos Guararapes	2010	*Lysinibacillus sphaericus*	4.3 (0.017)	–	S	AS-PCR	cqm1_REC_ (0.001)^e^; cqm1_REC-D16_ (0.003)^e^	Chalegre et al. [89]
			2010	*Lysinibacillus sphaericus*	–	–	–	Multiplex PCR	cqm1_REC_ (0.003)^e^; cqm1_REC-2_ (0.002)^e^	Menezes et al. [85]
			2009–2010	Temephos	0.57 (0.002)	–	S	–	–	Amorim et al. [51]
		Glória do Goitá Ipojuca	2009–2010	Temephos	1.5 (0.009)	–	S	–	–	Amorim et al. [51]
			2009–2010	Temephos	0.6 (0.0019)	–	S	–	–	Amorim et al. [51]
			2010	*Lysinibacillus sphaericus*	3.3 (0.013)	–	S	AS-PCR	cqm1_REC_ (0.003)^e^	Chalegre et al. [89]
		Santa Cruz do Capibaribe	2009–2010	Temephos	5.8 (0.036)	–	Moderate resistance	Enzymatic assays and AS-PCR	G119S ace-1^R^ (0.11)^e^; α-esterase (> 30%); β-esterase (> 17%)	Amorim et al. [51]
	Ceará	Fortaleza	1993	–	–	–	–	Enzymatic assays	Esterase C2	Yebákima et al. [60]
Center-West	Mato Grosso	Cuiabá	2000	Temephos	–	0.012–0,06 ppm (95–100%)	Tolerant	–	–	Campos & Andrade [50]
	Mato Grosso do Sul	Campo Grande	1998	Temephos	–	0.04 ppm (88.09); 0.045 ppm (93.71%)	R	–	Metabolic resistance (suggested)	Campos & Andrade [50]
			2012	–	–	–	–	AS-PCR	L1014F kdr mutation (0.01)^e^	Steinhagem et al. [77]
		Naviraí	2013	Temephos	–	0.004 ppm (18–20%)	R	–	–	Scudeler et al. [52]
South-East	Rio de Janeiro	Rio de Janeiro	1978	Chlorpyriphos	2.2 (0.001)	–	Low resistance	–	–	Curtis & Pasteur [48]
			1994	Malathion	2.2 (0.15)	–	S	Sinergists tests	Mixed function oxidase and increased esterases	Gonzáles et al. [49]
				Chlorpyriphos	78.9 (0.0005)	–	R	–	Mixed function oxidase and increased esterases	
				Pirimiphos-methil	4.4 (0.026)	–	S	–	–	
				Propoxur	5.1 (0.51)	–	S	Sinergists tests	Mixed function oxidase and increased esterases	
				Cypermethrin	3.4 (0.0008)	–	S	–	–	
				Deltamethrin	3.2 (0.0003)	–	S	–	–	
				Lambda-cyhalothrin	6.0 (0.0003)	–	S	Sinergists tests	Mixed function oxidase and increased esterases	
				DDT	11.8 (0.025)	–	R	–	–	
			1995	Malathion	43.81 (0.609)	–	R	Enzymatic assays	Elevated esterase and altered AChE	Coto et al. [24]
			2012	–	–	–	–	AS-PCR	L1014F kdr mutation (0.04)^e^	Steinhagem et al. [77]
		Niterói	2012	–	–	–	–		L1014F kdr mutation (0.07)^e^	
	São Paulo	Pinheiro River	2005	*Lysinibacillus sphaericus*	0.6–2.85 (0.009–0.0454)	–	S	–	–	Andrade et al. [30]
			2006	*Lysinibacillus sphaericus*	5 (0.015)	–	S	–	–	Silva-Filha et al. [91]
			1995	Fenitrothion, malathion and propoxur	–	1% (47.5%); 5% (58.8%); 0.1% (59.4%)	R	–	–	Bracco et al. [53]
			1995	DDT and permetrin	–	4%; 0.25% (100%)	S	–	–	
			1995–1996	Fenitrothion, malathion and propoxur	11.20 (136.3)^c^; 3.33 (32.9)^c^; 3.01 (200)^c^	–	R	Enzymatic assays	G119S ace-1^R^ (0.12–0,17)^e^; α-esterase (> 11.2%)	Bracco et al. [54]
		Campinas	1999–2001	Temephos	6.36 (0.0076)	–	R	–	Metabolic resistance (suggested)	Campos & Andrade [50]
			1999–2001	Cypermethrin	–	0.0096 ppm (11.17%)	R	–	Metabolic resistance (suggested)	
			1999–2001	Cyfluthrin	–	0.002–0.03 ppm (0.42–47.5%)	R	–	Metabolic resistance (suggested)	
	Minas Gerais	Belo Horizonte	2012	–	–	–	–	AS-PCR	L1014F kdr mutation (0.04)^e^	Steinhagem et al. [77]
South	Rio Grande do Sul	Porto Alegre	1989–1991	*Lysinibacillus sphaericus* and *Bacillus thuringiensis* svar*. israelensis*	–	1250 mg/m^2^ (100%)	S	–	–	Ruas-Neto et al. [97]
			1989–1991	Tetramethrine/PBO	–	5 mg/m^2^ (97.87%)	S	–	–	
		Feliz	1989–1991	*Lysinibacillus sphaericus* and *Bacillus thuringiensis* svar*. israelensis*	–	1250 mg/m^2^ (100%)	S	–	–	
			1989–1991	Temephos and fenthion	0.86^b^ (7.4×10^3^) and 1.39^b^ (0.24)	–	S	–	–	

*Note*: LC50 and LC95 expressed in ppm i.a

^a^Recife Metropolitan Area

^b^Lethal concentrations plus standard errors calculated after log transformations and anti-log values between brackets

^c^Lethal time: LT_50_

^d^In accordance to the original reference

^e^Allelic frequence

*Abbreviation*: AS-PCR, allelic specific polymerase chain reaction

## Organophosphates and carbamates

First reports of organophosphate resistance in *Cx. quinquefasciatus* from Brazil came from Rio de Janeiro populations evaluated with chlorpyrifos in 1978 [48] and later in 1994 [49]. Resistance to the larvicide temephos was described in populations from Campinas (São Paulo State) [50], Santa Cruz do Capibaribe (Pernambuco State) and Campo Grande [51] and Naviraí [52] (Mato Grosso do Sul State). In addition, a population from Cuiabá (Mato Grosso State) collected in 2000 was classified as “tolerant”, while *Ae. aegypti* collected at the same site and year were susceptible to the insecticide [50]. Resistance to the carbamate propoxur was evidenced in *Cx. quinquefasciatus* collected in the region of Pinheiros River in the center of São Paulo, in 1995 and 1996 [53]. Also, in São Paulo, resistance was detected to both organophosphates malathion and fenitrothion [53, 54] and to malathion (RR_50_ of 43.81) in Rio de Janeiro [24].

High levels of resistance to malathion were also observed in *Cx. quinquefasciatus* from other Latin American countries such as Cuba (RR_50_ of 207.91 and 135.97) and to a lesser extent Venezuela (RR_50_ of 16.11). The authors considered that the intense employment of the OP themephos, malathion and propoxur and also pyrethroids, for the first dengue outbreaks in the 1980’s, contributed to control *Ae. aegypti*, however inducing resistance in *Cx. quinquefasciatus* to OPs and propoxur [55, 56]. In Brazil, resistance to temephos is currently disseminated in *Ae. aegypti* populations throughout the country which forced the National Dengue Control Programme to replace this chemical by Insect Growth Regulators (IGRs) compounds [25]. This scenario of disseminated resistance to temephos might as well be extended to *Cx. quinquefasciatus*. For example, more than 40,000 kg of temephos were applied in the city of Santa Cruz do Capibaribe alone against *Ae. aegypti* during 2007–2010 which was likely the source of pressure that selected resistant *Cx. quinquefasciatus* in the region [51].

Organophosphates and carbamates both target the acetylcholinesterase enzyme (AchE), causing the accumulation of the neurotransmitter acetylcholine in a synaptic shift, which inhibits the interruption of a nervous impulse and therefore, kills the insect. The metabolic enzyme participation also plays an important role in organophosphate resistance in *Culex*. For instance, a gene or a set of esterase genes suffered several duplications, causing the increase of their codified enzymes and consequently, more sequestration of the insecticide molecule [57–59]. The register of detoxifying enzyme quantification associated with insecticide resistance in *Cx. quinquefsciatus* from Brazil was noted in populations from Fortaleza [60], São Paulo [53], Santa Cruz do Capibaribe [51] and Rio de Janeiro [24, 49].

A single nucleotide polymorphism (SNP) in the acetylcholinesterase gene (*ace-1*) with the substitution of a Gly by a Ser in the 119 codon (G119S, *ace-1*^*R*^ allele) is a target site mechanism mostly displayed in *Culex* populations resistant to organophosphates. The same G119S SNP is also found in other insects, including *Anopheles* mosquitoes [61–63]. Other mutations (F290V and F331W) were also described in the *Culex ace-1* gene, also possibly related to resistance to organophosphates [64–67]. The G119S was the only *ace-1* mutation found in *Cx. quinquefasciatus* from Brazil, in the localities of São Paulo [53], Recife [64], Santa Cruz do Capibaribe [51] and Rio de Janeiro [68].

## Organoclhorines and pyrethroids

The organochlorine DDT and the pyrethroids act in the voltage-gated sodium channel (Na_V_) in the neuron membranes, prolonging its open state. This interaction results in a repetitive firing of the nervous impulse, leading the insect to involuntary muscle spasms, exhaustion and death, a phenomenon known as knockdown effect [69]. Organochlorine may also inhibit the gamma-aminobutyric acid (GABA)-gated chloride channel. This is the case of cyclodienes, such as dieldrin which antagonizes the effects of the GABA receptor by preventing chloride ions from entering the neurons, thus inhibiting the return to resting state after an impulse transmission. A classical mutation (A302S) in the GABA receptor induces resistance to dieldrin, thus being referred to as RDL in several insects including *Cx. quinquefasciatus* [70, 71]. No substitution in the GABA gene has been reported in populations from Brazil. Increase in the action of detoxifying enzymes is usually related to DDT- and pyrethroid resistance, especially GST and monoxygenases P450 classes [72, 73]. However, given the diversity of multiple genes in these classes, it is difficult to find the same specific molecular marker for metabolic resistance across distinct species or even among populations of a same species. In the case of the target-site alterations, however, the same SNPs are found in distinct species, as is the case of the substitution Leu by Phe in the 1014 site of the *Na*_*V*_ gene, known as the classical *kdr* (knockdown resistance) mutation [74].

There is one report of a *Cx. quinquefasciatus* population from Rio de Janeiro collected in 1994 resistant to DDT [49]. The use of this compound had been discontinued against mosquitoes since 1971 and finally prohibited in Brazil in 1985 [75]. Therefore, this could result from a persisted resistance selected by DDT itself or cross-resistance by the use of other compounds. Resistance to the pyrethoids cyfluthrin and cypermethrin was identified in a population from Campinas collected in 1999, whilst simultaneously collected *Ae. aegypti* were susceptible [50]. In addition, there was resistance to deltamethrin in a laboratory strain from Divinópolis [76]. Resistance to deltamethrin emerged and spread very rapidly in *Ae. aegypti* since its introduction by national campaigns against the dengue vector in 2000. One decade later, high levels of resistance to this chemical were acquired throughout the country, especially during dengue epidemic seasons, probably with an important contribution of the use of household insecticide sprays, all pyrethroid-based products [25, 41]. This environment with constant insecticide application near and inside the houses has likewise been selecting resistance to pyrethroids in *Cx. quinquefasciatus*.

To date, there has only been one report for a *kdr* mutation in *Cx. quinquefasciatus* from Brazil in which the classical L1014F was detected, yet under low frequencies (4–7%) in samples from Campo Grande, Rio de Janeiro, Niterói and Belo Horizonte [77]. In these localities, resistance to deltamethrin was apparent in *Ae. aegypti* probably related to *kdr* mutations [78]. Similarly, L1014F was detected in *Cx. quinquefasciatus* from Mexico, indirectly exposed to pyrethroids targeting *Ae. aegypti* during dengue control campaigns [79]. Other substitutions were found in the same 1014 aminoacid position (L1014S, L1014C) in the *Cx. pipiens* complex [80]. However, none were ever observed in *Cx quinquefasciatus* from Brazil, other than L1014F.

## Biolarvicides

*Bacillus thuringiensis* var. *israelensis* (*Bti*) and *Lysinibacillus sphaericus* (*Lbs*) are bacteria that produce endotoxins that are activated by mosquito larvae intestinal proteases and bind to specific receptors in the intestinal epithelium, causing degeneration and consequently, killing the larvae [81, 82]. The *Lbs* is more suitable for controlling *Cx. quinquefasciatus* because it presents higher activity in polluted water when compared to *Bti* [83]. The *cqm1* gene encodes an epithelial protein to which the toxin binds. Some mutations in the *cqm1* gene were associated with *Lbs* resistance. For example, a deletion of 19 nucleotides (*cqm1*_*REC*_) and the substitution of a guanine (G) to an adenine (A) in the codon 1324 (*cqm1*_*REC-2*_), both present in *Cx. quinquefasciatus* field populations from Recife [84, 85]. Resistance to *Lbs* was found in a *Cx. quinquefasciatus* populations from Coque, an urban area of Recife, only two years after its implementation in 1991, reaching a resistance ratio 10-fold higher than the susceptible control [86]. However, this resistance was later reversed with the interruption of the biolarvicide application in that locality [87]. In other areas of Recife city, *Lsb* continued to be enlisted and several studies have been evaluating the levels of susceptibility to this bioinsecticide in the city as well as identifying new molecular markers [88–90]. These studies have shown that the frequency of resistant individuals in Recife city remains at low levels, even with the continued application of the bacterial insecticide, either alone or in combination with other larvicides such as *Bti* [91–94]. In a recent study in Colombia, *Lbs* proved to be efficient against both *Cx. quinquefasciatus* and *Ae. aegypti* field populations, suggesting it as an interesting alternative to chemical insecticides [28].

## Conclusions

In accordance with the WHO Global Vector Control Response [95], the emergence and spread of vector-transmitted diseases is likely to be intensified in the following years, especially those with the participation of urban mosquitoes such as *Culex* spp., given their strong adaptation to climate changes and inefficient urban sanitary infrastructures. Therefore, as the application of insecticides is a primarily action against *Cx. quinquefasciatus* in Brazil, it is urgent to investigate the status of susceptibility/resistance of natural populations to all the chemical compounds available for use. Effective vector control in Brazil is a complex and multifactorial task considering the continental dimensions together with the greatly heterogeneous ecological and demographic aspects [96]. Future successful campaigns based on the use of chemicals have to implement a constant monitoring of insecticide effectiveness, employing integrated methods against all targeted species and considering a plan well adapted to regional peculiarities.

## Data Availability

Not applicable.

## Abbreviations

DDT: dichlorodiphenyltrichloroethane
*ace-1*: acethylcholinesterase 1 gene
AchE: acethylcholinesterase enzyme
*Na*_*V*_: voltage-gated sodium channel gene
WHO: World Health Organization
IRS: insecticide residual spraying
MoH: Ministry of Health
IGR: insect growth regulator
RR: resistance ratio
OP: organophosphate
SNP: single nucleotide polymorphism
*Kdr*: knockdown resistance
